# Concurrent bandgap narrowing and polarization enhancement in epitaxial ferroelectric nanofilms

**DOI:** 10.1088/1468-6996/16/2/026002

**Published:** 2015-04-08

**Authors:** Marina Tyunina, Lide Yao, Dagmar Chvostova, Alexandr Dejneka, Tomas Kocourek, Miroslav Jelinek, Vladimir Trepakov, Sebastiaan van Dijken

**Affiliations:** 1 Microelectronics and Materials Physics Laboratories, University of Oulu, PO Box 4500, FI-90014 Oulun Yliopisto, Finland; 2 NanoSpin, Department of Applied Physics, Aalto University School of Science, PO Box 15100, FI-00076 Aalto, Finland; 3 Institute of Physics, Academy of Sciences of the Czech Republic, Na Slovance 2, 182 21 Prague 8, Czech Republic; 4 Ioffe Physical-Technical Institute RAS, 194021 St. Petersburg, Russia

**Keywords:** epitaxial growth, ferroelectric nanofilms, optical properties

## Abstract

Perovskite-type ferroelectric (FE) crystals are wide bandgap materials with technologically valuable optical and photoelectric properties. Here, versatile engineering of electronic transitions is demonstrated in FE nanofilms of KTaO_3_, KNbO_3_ (KNO), and NaNbO_3_ (NNO) with a thickness of 10–30 unit cells. Control of the bandgap is achieved using heteroepitaxial growth of new structural phases on SrTiO_3_ (001) substrates. Compared to bulk crystals, anomalous bandgap narrowing is obtained in the FE state of KNO and NNO films. This effect opposes polarization-induced bandgap widening, which is typically found for FE materials. Transmission electron microscopy and spectroscopic ellipsometry measurements indicate that the formation of higher-symmetry structural phases of KNO and NNO produces the desirable red shift of the absorption spectrum towards visible light, while simultaneously stabilizing robust FE order. Tuning of optical properties in FE films is of interest for nanoscale photonic and optoelectronic devices.

## 1. Introduction

Optical and photonic devices have long been employing strong optoelectronic effects [1, 2], nonlinear optical properties [3], acousto-optic and thermo-optic effects [4], and photostriction of perovskite ferroelectric (FE) crystals [5]. Since FE polarization enables the separation of photo-induced charge carriers, very large photovoltages and efficient photocatalysis can be achieved [7–11]. The potential of such effects for clean energy and environment applications using solar light has renewed interest in FEs and stimulated research on innovative methods to narrow their bandgap [12, 13]. Besides, the interaction of light with FEs is envisaged to lead to new, yet undiscovered phenomena and applications [14]. The synthesis of FE materials at the nanoscale is also of great interest for applications in integrated optics and photonics. In particular, optical probes based on second harmonic generation in FE nanowires and nanoparticles have been developed for sub-wavelength imaging in biological and clinical applications [15, 16]. Efficient photovoltaic structures [17], photovoltaic-based memory devices [18], high-bandwidth optoelectronic modulators [19], and tunable plasmonic devices using epitaxial FE films have also been demonstrated [20]. Additionally, the integration of optically active FE films with silicon photonics has become feasible [21]. A desirable lowering of the operating voltage of such devices requires a reduction of the FE film thickness. Interestingly, for a thickness of only a few nanometers, electric fields as large as 10^8^–10^9^ Vm^−1^ are already obtained by the application of 1–10 V. Such fields are comparable to the intra-atomic electric field of FEs, which is anticipated to generate nonlinear effects and additional functionalities.

Compared to single-crystal prototypes, the crystal structure of heteroepitaxial nanofilms can be substantially changed. For instance, epitaxial growth may result in the formation of new structural phases with electronic and optical properties that do not exist in bulk crystals. Another, much more established effect in heteroepitaxial systems is strain, which is caused by a mismatch between the lattice parameters of the substrate and film material or a difference in thermal expansion coefficient. It is well known that strain affects the bandgap and optical properties of semiconductors. Strain-controlled tuning of the bandgap in semiconductors is now a textbook example [22], which in practice is used for the engineering of optoelectronic properties in semiconductor heterostructures and novel 2D nanomaterials, including free-standing membranes [23, 24]. Typically, biaxial in-plane strain reduces the bandgap of semiconductors by 60–100 meV per 1% strain. The same relationship between lattice strain and bandgap has been found for epitaxial nonpolar films of simple metal oxides [25]. In contrast, a polarization-induced bandgap widening dominates in FE materials, which can be described by a simple phenomenological model [26]. Normally, the crystal symmetry is lowered (e.g. from cubic to tetragonal) when FE polarization (*P*) is stabilized at the paraelectric–ferroelectric (PE–FE) phase transition. Compared to the PE phase, the bandgap of the FE phase widens by *ΔE_g_* [26]:1
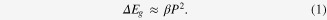


The validity of this relationship with coefficient *β* ≈ 1 (if the energy is expressed in eV and polarization is expressed in Cm^−2^) has been confirmed experimentally for FE crystals [27, 28]. The dominating effect of polarization on the width of the bandgap has been evidenced also by measurements on epitaxial BiFeO_3_ films. In this case, the bandgap widens by approximately 0.4 eV when the polarization increases from 0.9 Cm^−2^ in the rhombohedral phase to 1.5 Cm^−2^ in tetragonal films [29–31]. Moreover, compared to an indirect bandgap of 3.29 eV in bulk crystals of quantum PE SrTiO_3_ (STO), an indirect bandgap of 3.82 eV has been found for epitaxial FE STO films with 2% lattice strain [32–34]. The experimental results on STO agree with first-principles (FPs) calculations [35], showing that the effect of FE polarization on the bandgap dominates over the effect of strain. In this picture, strain-enhanced FE polarization leads to an effective widening of the bandgap [36] and bandgap narrowing seems impossible in the FE state.

Here, we report on versatile engineering of the electronic band structure in FE nanofilms of KTaO_3_ (KTO), KNbO_3_ (KNO), and NaNbO_3_ (NNO) with a thickness of 10–30 unit cells. KTO, KNO, and NNO crystals are end members of solid solutions with excellent piezoelectric properties and unique diffractionless light propagation and KNO crystals are widely used as nonlinear optical material [37–39]. We find that the bandgap of epitaxial FE nanofilms can be reduced in the presence of enhanced polarization when the structural symmetry of the film is increased compared to that of the bulk crystal.

## 2. Experiment

KTO, KNO, and NNO films with a thickness of 10–30 unit cells were grown using ablation of ceramic targets stimulated by radiation from a pulsed KrF excimer laser. The fluence of the incident radiation was ∼2 Jcm^−2^ and the repetition rate of the laser pulses was 5 Hz. The STO substrates were placed onto a resistively heated holder in on-axis substrate-target configuration. During deposition, the substrate temperature was 973 K and the pressure of high purity oxygen was 20 Pa. The oxygen pressure was raised to 800 Pa during post-deposition cooling, which was conducted at a rate of 5 K min^−1^. To ensure film stability during cross-sectional specimen preparation for transmission electron microscopy, heterostructures with a thin protective top layer of STO were also grown.

The crystal structure and the lattice strains of the FE nanofilms were analyzed using high-resolution transmission electron microscopy (HRTEM). The samples for cross-sectional HRTEM were prepared by standard techniques. High-resolution imaging was carried out in a JEOL 2200FS TEM with double Cs correctors, operated at 200 kV. A FRWR-tools plugin for Gatan Digital Micrograph software was used to perform geometric phase analysis (GPA) [44]. The maps of the in-plane and out-of-plane strain were calculated from the HRTEM images using the lattice of the STO substrate as a reference. In the calculations, a Gaussian filter to selected pairs of (101) and (10-1) reflections in the fast Fourier transform pattern was used. The spatial resolution of the GPA maps is determined by the choice of filter size. For all mappings in this study, the resolution was set to 0.6 nm. The chemical composition of the films was inspected using energy-dispersive x-ray spectroscopy. Proper cation stoichiometry was confirmed for all films.

The optical properties of uncovered films and bulk crystals were probed using variable-angle spectroscopic ellipsometry (VASE), which allows for high-precision measurements of films with a thickness of only a few atomic layers and of separate macromolecules on top of arbitrary substrates [45]. Since accurate analyses of ellipsometric data for wide bandgap materials are only possible if the spectral range is sufficiently expanded into the high-energy (UV) region, an J A Woollam ellipsometer with an extended photon energy range was used. Room temperature ellipsometric data were collected using a variable-angle rotating-analyzer spectroscopic ellipsometer over a spectral range from 0.74 to 9.0 eV. Spectra of ellipsometric angles (*Δ*, *ψ*) were extracted with an accuracy of 0.2^°^ for *Δ* and 0.04° for *ψ* by measuring each sample at two angles of incidence (*Θ* = 65°, 70°) [46]. VASE data analysis was performed using the WVASE32 software package [47]. The experimental ellipsometric spectra were fitted using a model in which a stack of a semi-infinite substrate, a FE film, a surface roughness layer, and ambient air is considered. The parameterization of the initial dielectric functions of the films was based on the multi-oscillator model. The optical properties of the surface roughness layer were represented by a Bruggeman effective medium approximation [48]. The initial optical spectra and thickness of the FE film and surface roughness layer were extracted using a least-square regression analysis. After a refinement of the initial optical properties and layer thickness, the thickness was fixed and numerical inversion was used to extract the complex refractive index as a function of photon energy. The absorption coefficient *α* was obtained using the relationship *α* = 4*πk*/*λ*, where *k* is the extinction coefficient (the imaginary part of the complex index of refraction) and *λ* is the wavelength of the light. The VASE measurements ensured accurate and reliable data for the absorption coefficient down to a few hundred cm^−1^.

The dielectric functions and the optical properties of bulk STO substrates and NNO, KTO, KNO crystals were determined from separate measurements on bulk samples. The data analysis accounted for the surface roughness layer. Several STO substrates from the same batch (MTI Corp.) were studied as a reference. Their optical properties were found to be similar and were used in VASE data processing. The mean square error for the nanofilms was in the range of 0.1–0.25, demonstrating high reliability [49].

## 3. Results and discussion

**Figure 1.**
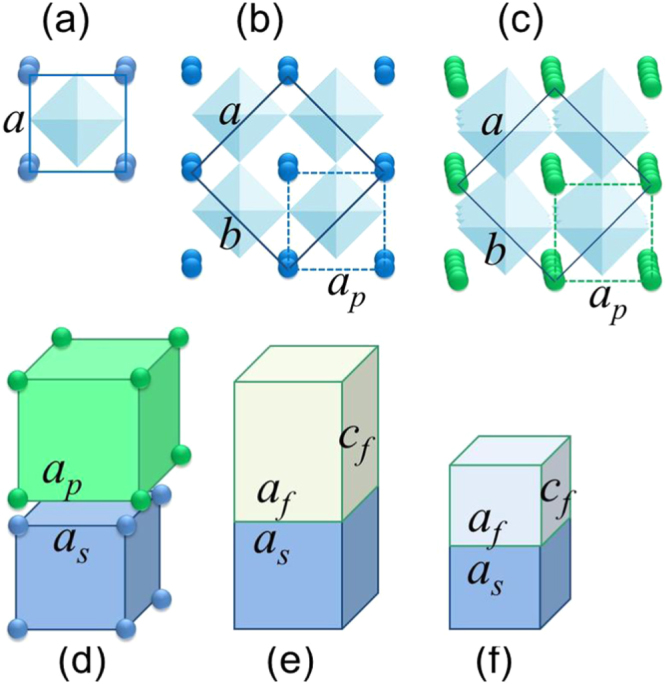
Crystal structure and coherent epitaxy of perovskite films. (a) Cubic perovskite unit cell. Spheres represent *A*-site ions and the *B*O_6_ octahedron is shown in light-blue. Orthorhombic unit cell (marked by solid line in *a*–*b* plane) and perovskite cell (dashed line) of KNO (b) and NNO (c) bulk crystals. (d) Lattice misfit for cube-on-cube growth of perovskite films on STO (001). (e) Tetragonal structure of epitaxial films with in-plane compressive misfit strain at the deposition temperature. (f) Same structure as in (e) after cooling down to room temperature.

At room temperature, the bulk crystal structure of KTO is cubic (figure 1(a)) and the bulk lattices of KNO (figure 1(b)) and NNO (figure 1(c)) are orthorhombic, whereas all materials (KTO, KNO, NNO, and STO) possess a cubic crystal structure at *T*_dep_ = 973 K [40]. During cube-on-cube epitaxy of KTO, KNO, and NNO on STO (001) substrates, the in-plane lattice parameters of the perovskite cell are compressed for all films (figure 1(d)). The in-plane biaxial misfit strain *s* in the films, here defined as *s* = (*a*_*s*_/*a*_*p*_ − 1) with *a*_*p*_ and *a*_*s*_ indicating the bulk lattice parameters of the perovskite film material and the STO substrate, is about −2% in KTO, −3% in KNO, and –1% in NNO. As a result, the films grow with a metrically tetragonal structure (figure 1(e)) up to a critical thickness, beyond which the strain starts to relax [41]. Upon cooling to room temperature, the film strain changes due to a mismatch between the thermal expansion coefficient of the film and substrate (figure 1(f)). The resulting room temperature crystal structures, biaxial in-plane lattice strains, and electric phases of ideal cube-on-cube epitaxial KTO, KNO, and NNO nanofilms on STO (001) are summarized in table 1. Compared to bulk crystals, novel structural and FE phases form in epitaxial films on STO [34, 42], namely the FE tetragonal *c*-phase with out-of-plane polarization along the [001] crystal direction in KTO (*s* = −2.1%) and KNO (*s* = −2.8%) and the FE pseudo-cubic *r*-phase with polarization along the out-of-plane [001] and in-plane [110] directions in NNO (*s* = 0%). Thus, compared to bulk crystals, the structural symmetry is lowered in nanofilms of KTO [43] and it is enhanced in KNO and NNO.

**Table 1.** Comparison of crystal structure and electric phase (paraelectric (PE), ferroelectric (FE), antiferroelectric (AFE)) of KTO, KNO, and NNO in bulk crystals and in ideal epitaxial films on STO (001) at room temperature. Here, *a*_pc_ indicates the lattice parameter of the pseudo-cubic perovskite unit cell.

Material	Bulk				Thin film		
	Structure	Phase	Lattice parameter (Å)	*a*_pc_ (Å)	Strain (%)	Structure	Phase
KTaO_3_	cubic	PE	*a* = 3.988	3.988	−2.1	tetragonal	*c*-FE
			*a* = 5.697				
KNbO_3_	orthorhombic	FE	*b* = 5.723	4.016	−2.8	tetragonal	*c*-FE
			*c* = 3.971				
			*a* = 5.506				
NaNbO_3_	orthorhombic	AFE	*b* = 5.566	3.903	0.0	pseudo-cubic	*r*-FE
			*c* = 15.520				

### 3.1. Structural characterization

**Figure 2.**
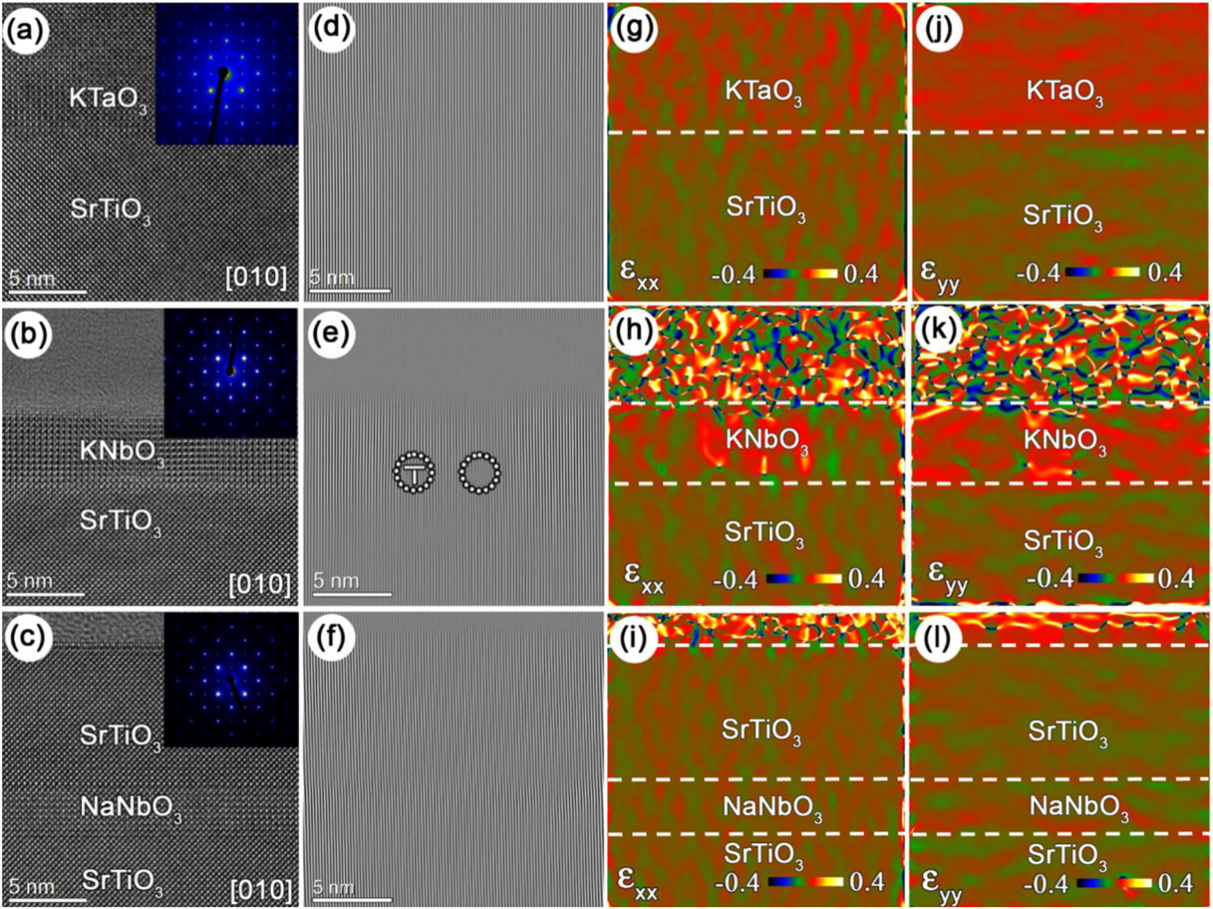
HRTEM images and lattice strains of epitaxial KTO, KNO, and NNO nanofilms on STO (001). (a)–(c) Cross-sectional images along the [010] zone-axis. SAED patterns are shown in the insets. (d)–(f) (200) Fourier filtered images corresponding to the HRTEM images of (a)–(c). (g)–(i) In-plane strain (*∊*_*xx*_) and (j)–(l) out-of-plane strain (*∊*_*yy*_) maps based on the HRTEM images of (a)–(c). The STO substrate lattice was used as a reference in the strain calculations. The white dashed lines in (g)–(l) indicate the interfaces between the layers.

HRTEM analyses confirm epitaxial growth of KTO, KNO, and NNO films with a thickness of 10–30 unit cells on top of STO (figure 2). Selected area electron diffraction (SAED) patterns evidence (001)[100]film||(001)[100]STO cube-on-cube epitaxy. Fourier filtered images do not show any misfit dislocations in KTO and NNO suggesting fully coherent growth of the KTO and NNO films on STO. Dislocations are not formed in KTO up to a film thickness of at least 10 nm. On the other hand, random misfit dislocations are detected in KNO, indicating local strain relaxation.

The spatial distribution of lattice strain is visualized by GPA of the HRTEM images (figures 2(g)–(l)). The in-plane strain maps of KTO and NNO exhibit uniform contrast, which implies excellent film-substrate lattice coherency with equal in-plane lattice parameters for the films and STO substrate. The in-plane KNO–STO lattice coherency is good also apart from a few dislocations. Although the strain locally relaxes by the formation of misfit dislocations, a regular network of dislocations is not formed. As a result, most of the KNO film remains strained. The out-of-plane strain maps demonstrate pronounced film-substrate contrast for KTO and KNO with uniform strain distributions along the growth direction in KTO and some disruptions around the dislocations of KNO. The out-of-plane lattice parameters of KTO and the strained fraction of KNO are about 3% larger than the lattice parameter of the STO substrate, which is consistent with their tetragonal lattice structure. The out-of-plane strain map of NNO evidences coherent growth of NNO on STO with equal out-of-plane lattice parameters for NNO and the STO substrate.

### 3.2. Optical properties

**Figure 3.**
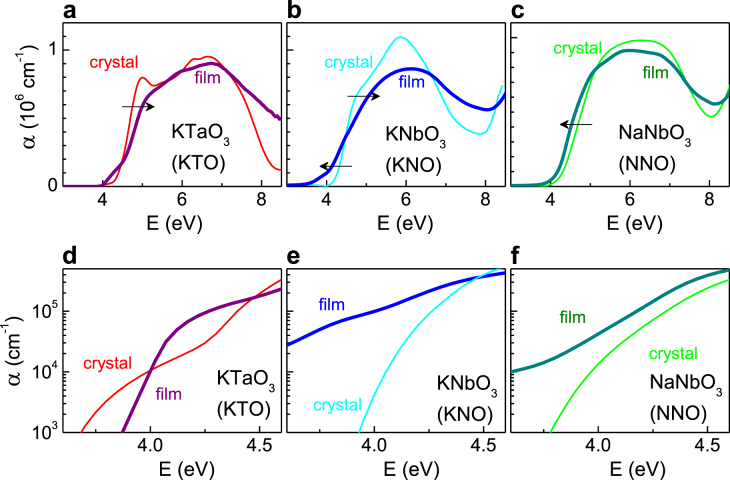
Absorption coefficient *α* as a function of photon energy *E* for bulk crystals (thin curves) and epitaxial nanofilms (thick curves) of KTO (a), (d), KNO (b), (e), and NNO (c), (f). The arrows in (a)–(c) indicate the spectral shifts. Note that data for *α* near the absorption edge are plotted on a logarithmic scale in (d)–(f).

**Figure 4.**
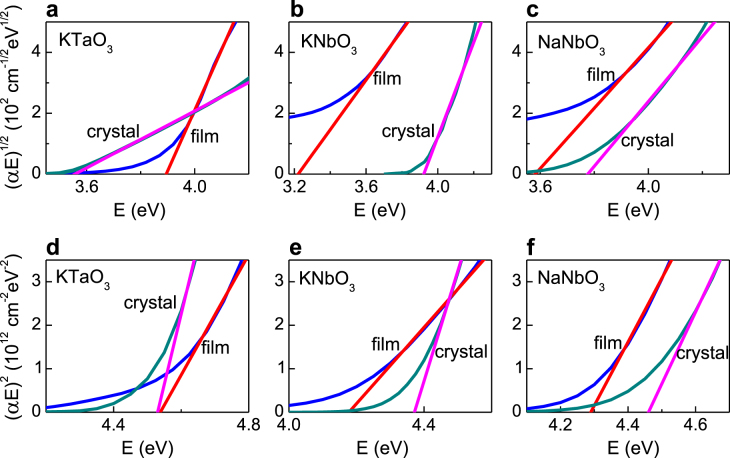
Tauc plots for indirect (a)–(c) and direct (d)–(f) transitions in nanofilms and bulk crystals of KTO (a), (d), KNO (b), (e), and NNO (c), (f). The straight lines are linear fits to the data.

The optical absorption coefficient *α* as a function of photon energy *E* for the epitaxial FE nanofilms and bulk crystals of KTO, KNO, and NNO are shown in figure 3. The energies of the near-bandgap transitions were analyzed using linear fits to Tauc plots (*αE*)^1/2^ ∝ (*E − E*_i_) and (*αE*)^2^ ∝ (*E − E*_d_) for indirect and direct transitions (figure 4). For a film thickness of 4–10 nm, the optical properties did not vary. The extracted data are summarized in table 2. The epitaxial nanofilms and bulk crystals exhibit main absorption maxima at energies in the range of 5–7 eV. The indirect bandgaps and direct transitions are found at *E*_i_ = 3.2–4.05 eV and *E*_d_ = 3.6–5 eV. The observations for bulk crystals are consistent with earlier reports [40]. Compared to these reference crystals, the energies of the absorption maxima and near-bandgap transitions of the nanofilms are clearly different. The change in the absorption coefficient near the absorption edge amounts one to two orders of magnitude for all films. Importantly, the changes occur above an optical absorption of more than 1000 cm^−1^. Since oxygen vacancies and other defects typically affect the absorption by less than 100 cm^−1^ [50, 51], they can be ruled out as the main source of the effect. The photon energy *E*_*α*_ for which the absorption coefficient equals *α* = 10^4^ cm^−1^ is used additionally for the quantification of optical differences between the thin films and bulk crystals.

**Table 2.** Energies of the absorption maxima (*E*_m_), direct transitions (*E*_d_), indirect bandgaps (*E*_i_), and onset of *α* = 10^4^ cm^−1^ (*E*_*α*_) in bulk crystals and epitaxial nanofilms. The asterisk denotes a broad absorption maximum.

Material	KTaO_3_		KNbO_3_		NaNbO_3_	
	Bulk	Film	Bulk	Film	Bulk	Film
E_m_ (eV)	6.65 ± 0.01	6.75 ± 0.01	5.85 ± 0.01	6.12∗± 0.02		6.7∗± 0.1
	6.3 ± 0.01	6.0∗ ± 0.03	4.8∗ ± 0.1		6.28∗± 0.03	6.0∗± 0.1
	5.5∗ ± 0.03					
	5.0 ± 0.01	5.1∗ ± 0.04				5.1∗± 0.1
E_d_ (eV)	4.15 ± 0.02	4.55 ± 0.02	4.38 ± 0.02	4.18 ± 0.02	4.45 ± 0.02	4.29 ± 0.02
E_i_ (eV)	3.55 ± 0.02	3.9 ± 0.02	3.95 ± 0.02	3.21 ± 0.02	3.78 ± 0.02	3.56 ± 0.02
E_*α*_ (eV)	3.98 ± 0.01	4.00 ± 0.01	4.05 ± 0.01	3.08 ± 0.02	3.97 ± 0.01	3.61 ± 0.01

For KTO, the absorption maxima, bandgap, and near-bandgap transitions of the film are shifted to higher energies. Also, the absorption spectrum of the KTO nanofilm is considerably smoother compared to that of the bulk crystal. The first feature is in line with the expected effects of strain-induced FE polarization. Considering a difference of *ΔE* ≈ 0.4 eV between the energies of the near-bandgap transitions in the epitaxial film and the bulk crystal (table 2), the strain-induced polarization *P* of the KTO nanofilm can be estimated from equation (1). We find *P* ≈ 0.6 Cm^−2^, which agrees well with FPs calculations [34]. Besides this phenomenological argument, bandgap widening by FE polarization in *AB*O_3_ perovskites is also explained by microscopic mechanisms. The top of the relatively flat valence band in such FEs is formed by oxygen 2p orbitals that are hybridized with d orbitals of *B*-site ions. The conduction band edge at the *Γ* point mainly consists of *B*-site d*∊* states. FE polarization splits the conduction band levels and raises their energy, while the top of the valence band is only weakly affected [27, 28], i.e. FE polarization widens the bandgap. Spectral smoothing in thin films is caused by mixing of *B*-site 3d and O 2p states in the FE phase [27, 28, 34, 35]. Our data for KTO confirm the predicted effects of FE polarization and suggest that it is impossible to reduce the bandgap by an increase of lattice strain. Next, we will demonstrate that significant bandgap narrowing can still be achieved in FE nanofilms by an increase of structural symmetry.

For the KNO nanofilm (figure 3(b)), a smoothing of the spectral features and a blue shift of the main absorption maxima with respect to the KNO bulk crystal are found. These changes can be ascribed to the effect of FE polarization, which is enhanced by lattice strain to about 0.5 Cm^−2^ in the nanofilm compared to about 0.3 Cm^−2^ in the bulk crystal [40, 42]. However, instead of a corresponding polarization-induced bandgap widening, a strong increase of optical absorption at *E* < 4 eV and a red shift of the indirect bandgap and direct transition are measured. Contrary to KTO, epitaxial growth of KNO on STO increases the structural symmetry (the structure of KNO changes from orthorhombic in bulk to tetragonal in nanofilms, see table 1). The reduction of the bandgap is significant with *E*_i_ decreasing by as much as 0.74 eV.

The influence of lattice symmetry is further explored by measurements on an epitaxial film of NNO whose pseudo-cubic phase is of higher symmetry than the bulk orthorhombic structure. Remarkably, red shifts of the absorption maxima and of the indirect bandgap occur in the NNO film in the absence of lattice strain (*s* = 0%) and the presence of FE polarization (figure 3(c)) [28, 42]. Compared to the absence of FE polarization in NNO bulk crystals, a polarization of up to 0.3 Cm^−2^ is stabilized in NNO films [42, 52], which according to equation (1) would correspond to a bandgap widening of 0.1 eV. The observed reduction of the bandgap by 0.2 eV thus contrasts with the expected polarization-induced blue shift. Additionally, the spectrum of the NNO nanofilm is more structured. Just like the KNO nanofilm, the changes in the electronic transitions of NNO are against FE polarization-related effects.

**Figure 5.**
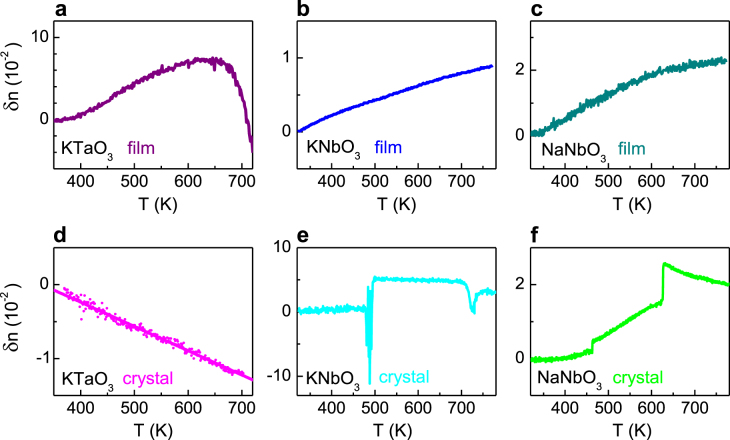
Variation of index of refraction *δn* at *E* = 2 eV as a function of temperature *T* in epitaxial nanofilms (a)–(c) and bulk crystals (d)–(f) of KTO (a), (d), KNO (b), (e), and NNO (c), (f). Here, the temperature variation *δn* of the index *n*(*T*) is defined as *δn* = *n*(*T*) − *n*(*R*), where *n*(*R*) is the room temperature value.

Our results suggest that new structural phases of epitaxial nanofilms are primarily responsible for complex changes in the electronic band structure. Most significantly, these include bandgap narrowing in the presence of robust FE polarization for structural phases of KNO and NNO with higher symmetry than their bulk crystals. Enhancements of the FE phases in the KTO, KNO, and NNO nanofilms are confirmed by the temperature evolution of the index of refraction (*n*(*T*)) as shown in figure 5. The index *n*(*T*) of the KTO nanofilm increases on cooling at high temperatures, which is typical for the PE state [53] and qualitatively similar to the behavior of the fully PE KTO bulk crystal. The decrease of *n* below 650 K, however, evidences the presence of FE polarization [34, 53]. The thermo-optical behavior of NNO and KNO nanofilms also differs from that of their bulk crystals. The PE–FE phase transition occurs at 725 K in the KNO crystal as manifested by an anomaly in the *n*(*T*) curve. The non-monotonic positive slope of *n*(*T*) for the KNO nanofilm indicates a stable FE state up to at least 780 K (the maximum temperature in this measurement). This upward shift of the FE phase transition is in qualitative agreement with the strain–temperature phase diagram of epitaxial perovskite FE films [54]. The structural phase transition, indicated by an anomaly of *n*(*T*) around 485 K in the KNO crystal, does not exist in the tetragonal KNO nanofilm. An absence of bulk-like phase transitions has also been found for FE BaTiO_3_ films [54]. The presence of a stable FE state with high transition temperature (>780 K) and the absence of distinct phase transitions at low temperatures are also found for the NNO nanofilm, which again differs considerably from the characteristics of the NNO bulk crystal. The data of figure 5 thus clearly indicate an enhancement of the FE phase in all three nanofilms.

The observation of bandgap narrowing in KNO and NNO nanofilms is against the effect of FE polarization. Strong correlations between the higher-symmetry structures of KNO and NNO nanofilms and bandgap narrowing on one hand, and an opposite effect (reduced structural symmetry + bandgap widening) for KTO on the other, suggest that new structural phases affect the electronic transitions more than FE polarization. A more detailed theoretical analysis of this anomalous bandgap behavior using FP calculations is difficult. While approaches based on density functional theory (DFT) are widely employed in the search for new materials with reduced bandgaps [55–57], their quantitative predictions are inaccurate for FE perovskites. Reasons for this include omissions of electron–photon interactions, the probability of electronic excitations, and, in many cases, FE polarization. In particular, the calculated bandgaps for KTO, KNO, and NNO are considerably smaller than experimental values and vary from work to work: 2.06–2.98 eV for KTO, 1.4–2.5 eV for KNO, and 1.63–2.33 eV for NNO (see [58–64] and references therein). Although FP analyses based on more sophisticated methods than DFT tend to give more realistic bandgaps (see [65–67] and references therein), such methodologies have not yet been developed for FE materials. It is worth noticing, however, that despite the mentioned difficulties and uncertainties in FP calculations, recent theoretical work has shown that off-center displacements of *B*-site cations may cause bandgap widening in rhombohedral perovskites compared to tetragonal ones [64]. This result supports qualitatively our experimental observations.

## 4. Conclusions

In summary, heteroepitaxial growth allows for the creation of perovskite FE nanofilms with new crystal phases and controllable lattice strain. The formation of new structural phases with a different electronic band structure compared to bulk crystal prototypes significantly modifies the optical properties. Bandgap alterations as large as 0.3–0.8 eV are inferred from our optical measurements and, depending on nanofilm material and structure, either a widening or narrowing of the bandgap is realized. Most significantly, the formation of higher-symmetry structural phases of NNO and KNO allows for considerable bandgap narrowing in the presence of enhanced FE polarization. We note that these promising results, which could raise the efficiency of photovoltaic, photostrictive, and photocatalytic systems operating under solar light, are still early in our understanding of bandgap behavior in FEs and that details related to structural symmetry need to be carefully examined in future experimental and theoretical studies.
